# Navigating the Same Storm but Not in the Same Boat: Mental Health Vulnerability and Coping in Women University Students During the First COVID-19 Lockdown in the UK

**DOI:** 10.3389/fpsyg.2021.648533

**Published:** 2021-04-30

**Authors:** Gabriela Misca, Gemma Thornton

**Affiliations:** School of Psychology, University of Worcester, Worcester, United Kingdom

**Keywords:** mental health, women students, COVID-19, coping, vulnerability

## Abstract

Having a mental health diagnosis in both general and student populations has been found to be a risk factor for negative coping and increased psychological distress during the COVID-19 pandemic. Drawing on a subset of data from a large contemporaneous research study, this report explores the experiences of 36 women students with and without reported pre-existing mental health diagnoses during the first UK lockdown, in spring 2020. Specifically, the data explored self-reported coping with the restrictions, with the abrupt move to online learning, and the loss of support; as well as students' perceived strengths and difficulties in balancing their student role with family roles such as being a partner and/or a parent. Students with a pre-existing mental health diagnosis reported higher levels of loneliness compared to a matched sample of non-students, and more avoidant coping and negative emotional coping than students without a diagnosis. Qualitative data illustrate how parenting intersects with well-being and stress as both a protective and risk factor for women university students. This research report adds to the evidence base on the impact of the COVID-19 pandemic on the student population, and how pre-existing mental health diagnoses intersect with coping behaviours and vulnerability in women students. Exploration of potential vulnerabilities can provide opportunities for targeted support, and identifying effective coping has the potential to inform effective interventions.

## Introduction

### Higher Education and Student Mental Health

According to the 2020 University Student Mental Health Survey (Pereira et al., 2020) prior to the COVID-19 pandemic, more than one in four (26.6%) students in UK Universities reported having a current mental health diagnosis. Furthermore, students with a prior mental health diagnosis and those who identify as female were at risk of further mental health decline in the absence of continued support after starting university (Pereira et al., 2020).

The number of students declaring a pre-existing mental health diagnosis to their university in the UK more than doubled since 2014/15 and, consequently, the vast majority (95%) of higher education providers in the UK reported an increase in demand for student counselling services (All Party Parliamentary Group on Students, 2020) and found themselves under increased pressure from government to provide more psychological and mental health support for students (Pereira et al., 2020). Widening participation in Higher Education (HE), led to increases in numbers of students from wider socioeconomic backgrounds. Concurrent decreases in government funding, increases in tuition fees, student loans, and reduced access to specialist mental health services created a situation of reduced support and increased financial pressures on students (Denovan and Macaskill, 2016). Student/staff ratios in UK Universities increased and support services have not kept pace with student numbers (Macaskill, 2013) leading to a reduction in available resources.

The unprecedented COVID-19 pandemic occurred in the context of these already existing concerns related to university students' mental health in general. This research report explores issues at the intersection between student mental health and the impact of the COVID-19 lockdown in the UK. Hence, the paper will firstly summarise the (emerging) studies reporting specifically on student mental health during the pandemic, and then will draw parallels with studies on the impact of the pandemic on mental health in the general population.

### Student Mental Health During the COVID-19 Pandemic

In addition to the academic and transitional stressors that university students ordinarily navigate, the COVID-19 pandemic brought novel and heightened challenges. Students have arguably suffered a 3-fold impact of the pandemic: their education has been disrupted by the abrupt change to remote online learning; social and peer group relationships have been negatively affected by reduced contact due to lockdowns and ensuing isolation; and graduate employment prospects were diminished due to the economic downturn brought by measures to control the virus's spread.

Struggling with the pandemic situation is not specific to students, but there are aspects of their experience which are uniquely challenging to navigate. Living away from home for the first time in their first year, being unable to build new friendships for social support and being prevented from travelling to visit their families can reasonably be understood to exacerbate feelings of loneliness and a sense of isolation (National Union of Students, 2020). Over half of UK university students reported that their mental health was in a worse state as a result of the COVID-19 situation (Hewitt, 2020). Only 20% of those struggling with their mental health during the pandemic reported seeking help (National Union of Students, 2020), suggesting that students who are in a position of vulnerability are also unable/unwilling to access support, or that accessing it is difficult. In 2018, Universities UK (Universities United Kingdom, 2018) developed a policy framework which advocates a holistic approach. This recommended that universities make mental health a priority and is thus seen as essential to successful university life. However, the low uptake in seeking support suggests that improvements are still needed.

Within the context of the pandemic, *the student experience* has been significantly affected. The COVID-19 pandemic has impacted students in different ways: for some, the greatest threat was infection with coronavirus; for others, the lockdown measures have been a dominating source of distress. Surveys of UK students since the beginning of the pandemic suggest high levels of stress and anxiety: 49% of students reporting they were “really worried” about loneliness and 42% being required to self-isolate (Save the Student, 2020); and 63% felt that COVID-19 was either a major or significant risk to their mental or physical health (Office for National Statistics, 2020).

Conversely, there are students who have benefitted from the changes to everyday life brought about by the pandemic: remote working and learning may have removed the logistical stressors such as travel costs and time. Individuals who struggle with the social aspects of education, may have benefitted from remote teaching and learning (Dvorsky et al., 2020), for example a survey of UK students, reported that more than half (59%) were very or quite satisfied with the online learning that has replaced face-to-face teaching (Hewitt, 2020).

The pandemic has posed challenges for all sectors of education and tested their organisational agility. The universities found themselves under pressure to prioritise changes that can be made effectively, within time constraints and cost/resource limitations. Universities around the world were in different positions in terms of preparedness for these forced changes. According to a report comparing 20 countries' higher education responses to the pandemic (Crawford et al., 2020) adaptations within UK higher education focused on Universities' ability to deliver curriculum content remotely during lockdown(s) and ensuring safety on campus. The priority to support students virtually without compromising academic quality, is consistent with some of the other countries, including China.

These preliminary findings on student mental health during the pandemic mirror the emerging trends from the general population. In order to enhance the understanding of the complex interplay between the pandemic and the pre-existence of mental health vulnerabilities and coping, the parallel findings from general population are considered below.

### COVID-19 Pandemic: Vulnerabilities, Mental Health, and Coping

Research in the UK reported higher than usual levels of stress, depression and anxiety in the general population during the COVID-19 pandemic (Jia et al., 2020). Such findings are not surprising when considered in the context of the multiple life-stressors that co-occurred in such an abrupt manner within a short period of time, including serious/life-threatening illness, bereavement, social distancing, and unemployment. All of these are considered to be significant threats to psychological health, and as such a degree of struggling to cope in these circumstances is to be expected (Jia et al., 2020; Polizzi et al., 2020). What is not yet clear, is whether the deleterious effects of the pandemic on mental health are a normal reaction to an unusual situation, and if adverse outcomes will abate when the crisis has passed, or whether they will endure and require intervention.

Consistent emerging findings indicate that vulnerability and inequality that existed prior to COVID19 have been exacerbated, resulting in those vulnerable beforehand suffering the most deterioration during the pandemic (Kousoulis et al., 2020; Pierce et al., 2020). Minority ethnic groups have suffered disproportionate COVID-19 mortality and morbidity (NHS, 2020). Women and young people are reported to have higher levels of anxiety and depression prior to and during the pandemic (Niedzwiedz et al., 2020; Fancourt et al., 2021). Other risk factors identified for worsened mental health are lower educational attainment, low income and pre-existing mental health diagnoses. Circumstances such as living alone or with children also increased risk (Fancourt et al., 2021). Losing access to in-person support and community resources as a result of imposed lockdowns impacts most those who rely on these sources of support to cope with daily life. Thus, people with pre-existing mental health diagnoses have been detrimentally affected by both the disruption to support services and their usual coping (Jia et al., 2020; Kousoulis et al., 2020).

Several UK reports indicate that mental health, when compared to pre-COVID levels, has deteriorated more in women (Daly et al., 2020) and parents of young children (Pierce et al., 2020; Cheng et al., 2021). Responsibilities such as homeschooling, shopping, food preparation, cleaning, informal care work, and maintaining relationship contacts, falls largely to women around the world (Power, 2020), which may account for increased stress in women during the pandemic (Cheng et al., 2021). Adults living with children report higher anxiety, but lower levels of depression (Niedzwiedz et al., 2020) and lone mothers are identified as a particularly vulnerable group (Xue and McMunn, 2020).

### Coping With the COVID-19 Pandemic

Coping responses modulate individual experiences of stress, determining affective and behavioural outcomes (Fluharty and Fancourt, 2020). Differing models of coping propose categorisation of coping behaviours as adaptive or maladaptive strategies, however the context is key in understanding the effectiveness of coping. For example, social support is recognised as a positive adaptive coping; however lockdown limited access to social support strategies by curtailing and reorganising social contacts. Specifically for students, this meant meeting fewer people, being confined with a small number of housemates, a return to their pre-university home or somewhere else.

Theoretical models (Stanisławski, 2019) categorise approaches to coping into *problem focussed coping* where individuals seek to address the cause of stress; and *emotion focussed coping* whereby they attempt to deal with the troubling emotions caused by stress. There is further delineation of strategies within these dimensions, for example, distinguishing between positive emotional coping and negative emotional coping (Stanisławski, 2019). An examination of the coping used during COVID-19 lockdown in a large scale UK study (Fluharty and Fancourt, 2020), found that women used active, problem and avoidant coping strategies more than men; and that young adults and people with pre-existing mental health diagnoses used more avoidant strategies (such as denial), but also that they turned more to supportive coping (accessing support services).

Similar trends were identified in populations across the globe. A report into coping behaviours across 16 countries identified the most frequent to be: watching television, social networking, listening to music, sleeping, domestic jobs, eating well, and completing unfinished work (Sameer et al., 2020). In Australia, females reported higher levels of distress than males, and positive coping such as reframing, acceptance, and humour were linked to better mental health (Gurvich et al., 2020). Conversely, negative coping behaviour such as avoidant strategies have been associated with increased distress, and were also negatively associated with well-being (Dawson and Golijani-Moghaddam, 2020). Moreover, negative coping strategies such as self-blame, venting, behavioural disengagement and self-distraction were linked to poorer mental health (Gurvich et al., 2020). Varma et al. (2021) found factors that negatively impacted successful coping were poor sleep, lower resilience and loneliness. They also report that younger age groups are particularly vulnerable to stress, anxiety, and depression and need more support. A study in Italy reported that a resilient coping style during the lockdown was negatively associated with depression, and specifically that engagement with active and flexible strategies of coping led to less depressive symptoms (Roma et al., 2020).

Shedding light on individual vulnerabilities provides opportunities for targeted support (Kousoulis et al., 2020). Identifying effective coping strategies has the potential to inform interventions to support individuals navigate the uncharted waters of the COVID-19 pandemic now and other global health crises in future. Crucially, understanding the link between pre-existing mental health diagnoses and increased psychological distress during the COVID19 pandemic will ensure support is targeted at the most vulnerable individuals.

### The Study Rationale

The “*same storm, different boats*” (Noonan, 2020) metaphor gained poignancy in discourses about how the pandemic has affected all lives across the board, while also shedding light onto individual differences and how these render some individuals more vulnerable, and some more able to cope and adapt. Emerging findings on mental health during the pandemic point toward deterioration throughout the population; but, importantly, that certain groups are more vulnerable: women, individuals with pre-existing mental health diagnoses and those living with young children. Individuals with the vulnerabilities identified in the general population are also represented in the higher education student population (The Higher Education Statistics Agency, 2020) and thus are likely to experience the compounded effects of the pandemic on their mental health. Although robust findings on the impact of the pandemic on students' mental health are yet to emerge, students are reporting that their mental health has been negatively affected (Pereira et al., 2020). However, there is limited understanding to date how individual vulnerabilities have contributed to the impact of the pandemic on student mental health.

This research report explores the experiences of women students during the first UK lockdown in the spring 2020, specifically: how they reported, retrospectively, their coping with the restrictions, the abrupt move to online learning, and with balancing their student role with family roles such as being a partner and/or a parent. The ultimate aim is to increase understanding of individual mental health vulnerabilities to the COVID-19 situation, which in turn can provide opportunities for targeted support, while distinguishing effective coping strategies has the potential to inform interventions.

## Method

This report focusses on a sample of UK women students (*N* = 36) enrolled in HE institutions in the UK during the first COVID-19 lockdown. The data for this report were extracted from a larger dataset gathered as part of a contemporaneous large-scale study, *Families Un-Locked* (Misca, 2020, 2021). This research study was launched in the UK August 2020 in partnership with Relate, the relationship charity (https://www.relate.org.uk/), and aimed to explore the medium and long-term effects of the pandemic related stressors on families and relationships. The research took an exploratory stance to understanding the impact of the lockdown and therefore employed a mixed method design, eliciting participants' reflections, and recollections of their behaviours and feelings during the first and strictest lockdown in the UK, which took place during March-June 2020. Data were collected post-lockdown, through a purposefully designed survey created specifically for this exploratory study. This included a combination of questions eliciting information about respondents' demographic and relationships in general and items aimed at the COVID-19 lockdown and post-lockdown context, assessing key variables related to family and relationships, coping, health, and well-being. Given the exploratory nature of the study and the unprecedented circumstance of the lockdown, the items' development was informed by the *Coping Circumplex Model* (Stanisławski, 2019) and the *Family Resilience Framework* (Walsh, 2016). As the data were collected *after* the first lockdown ended in the UK, it was therefore not feasible to use standardised measures of behaviours or mental states which occurred *during* the lockdown (as such measures usually rely on a 2–4 week retrospective time frame).

Research data analysed for the purpose of this report were collected during August-November 2020 and comprised participants' retrospective self-reports about their use of positive and negative coping (behaviours, feelings) during the first lockdown (March -June 2020) as compared to before the lockdown (pre-March 2020). Participants were asked to rate their coping *via* two multiple-questions sections, such as:

1. “During the COVID-19 lockdown, many people experienced lifestyle changes. Rate each item below indicating the changes you experienced compared to what it was like before lockdown”; responses to items were rated on a 3 point scale as *less, about the same, more*.
2. “During the COVID-19 lockdown, how have you been feeling?”; responses to items were rated on a 3 point scale: *most of the time, some of the time, not at all*)

The items addressed aspects such as (for details see Table 1):

- Positive coping—i.e.: spending more time on hobbies, doing exercise; positive emotional coping such as reframing; and positive social support connecting with friends/family remotely;
- Negative coping—i.e.: eating more, drinking more, having trouble relaxing, not feeling positive about the future.

**Table 1:** Coping during COVID-19 lockdown^a^.

	**Full student sample (*****N*** **=** **36)**		**PMHD**^****b****^ **students (*****n*** **=** **14)**		**NPMHD**^****3****^ **Students (*****n*** **=** **22)**	
	***n***	**%**	***n***	**%**	***n***	**%**
**Problem-focused coping**^**a**^						
*Positive*						
Going out (for walk, exercise)	15	42.9	5	35.7	10	47.6
Spending time on my hobbies	12	34.3	2	15,4	10	45.5
Reading	18	50	6	42.9	12	54.5
Cooking	20	55.6	6	42.9	14	63.6
Exercising	10	27.8	2	14.3	8	36.4
Keeping busy	7	19.4	3	21.4	4	18.2
*Social support—instrumental*						
Spending time connecting with family and friends on social media	15	41.7	6	42.9	9	40.9
Spending quality time with people in my household	20	55.6	9	64.3	11	50
*Avoidance*						
Drinking alcohol	12	33.3	5	35.7	7	31.8
Smoking	4	11.1	1	7.1	3	13.6
Eating	15	41.7	9	64.3^*^	6	27.3
Being bored	7	19.4	2	14.3	5	22.7
**Emotional coping**						
*Positive*						
Enjoyed having time for myself (most or some of the time)	30	83.4	11	78.5	19	86.3
Felt positive about the future (most or some of the time)	26	72.2	7	50	19	86.3^*^
*Social support—emotional*						
Enjoyed connecting with friends/family remotely *via* technology	8	22.2	3	21.4	5	22.7
Enjoyed spending more time with those I live with	17	47.2	5	35.7	12	54.5
*Negative*						
Felt anxious	10	27.8	6	42.9	4	18.2
Felt lonely	12	33.3	6	42.9	6	27.3
Had trouble relaxing	11	30.6	7	50^*^	4	18.2
Felt more tired than usual	19	52.8	9	64.3	10	45.5
Missed seeing family	11	30.6	4	28.6	7	31.8
Missed seeing friends	15	41.7	4	28.6	11	50

a *Reflects the number and percentage of participants answering “more [than before lockdown]” or “most of the time” [during lockdown].*

b *PMHD, Pre-existing Mental Health diagnosis.*

c *NPMHD, Non Pre-existing Mental Health diagnosis.*

* *p < 0.05 (for χ^2^-tests)*.

The impact of the pandemic on family relationships were assessed in two sections of the survey, one related to couple relationship and one to parenting.

- The *couple relationship* was assessed by 2 multiple set questions. One set of questions regarded couple behaviours: “How has the lockdown impacted your relationship?,” with answers rated on a 3 point scale (*less, about the same, more*), example items: “we have argued,” “we have supported each other” etc. The second set comprised statements rated on a 4 point scale (*strongly agree to strongly disagree*) on items such as “Things were bad already and the lockdown has made it worse,” ‘We are closer than before,” etc. (for details see Table 2).
- *Parenting practices* were assessed by a question: “During the lockdown how have you managed with the children?” rated on a 3-point scale (most of the time, some of the time, not at all) on items including being overwhelmed by childcare responsibilities; juggling childcare and work during lockdown, being anxious about their child's education, etc. (for details see Table 3).

**Table 2:** Couple relationships during COVID-19 lockdown.

	**Non-student couples (*****N*** **=** **36)**		**Student couples (*****N*** **=** **29)**		**PMHD**^****a****^ **students (*****n*** **=** **11)**		**NPMHD**^****b****^ **students (*****n*** **=** **18)**	
	***n***	**%**	***n***	**%**	***n***	**%**	***n***	**%**
**Couple relationship**^**c**^								
*Positive*								
Supported each other more as a couple	17	47.2	16	55.2	6	54.5	10	55.6
Talked more as a couple	12	33.3	12	41.4	4	36.4	8	44.4
Done things together more	19	52.8	10	34.5	3	27.3	7	38.9
Felt positive about our relationship	8	22.2	8	27.6	2	18.2	6	33.3
Felt close to partner	15	41.7	8	27.6	2	18.2	6	33.3
Lockdown has been a positive experience for us [agree or strongly agree]	18	50^*^	14	50	5	45.5	9	52.9
We are closer than we were before [strongly agree]	15	41.6	18	62.1	7	63.6	11	61.1
*Negative*								
We have argued more	14	38.9	9	31	4	36.4	5	27.8
We have grown apart	15	41.7	7	24.1	2	18.2	5	27.8
We have wound one another up more	15	41.7	9	31	4	36.4	5	27.8
Disagree on daily tasks more	7	19.4	9	31	5	45.5	4	22.2
We have felt more tension/strain	21	58.3^*^	12	41.4	7	63.6	5	27.8
We worried a lot about the pandemic causing tension in our relationship	12	33.4^*^						
Things were bad and lockdown has made it worse [strongly agree]	8	22.2	8	27.6	3	27.3	5	27.8
Lockdown put a real strain on our relationship [strongly agree]	16	44.5	14	48.3	5	45.5	9	50

a *PMHD, Pre-existing Mental Health diagnosis.*

b *NPMHD, Non Pre-existing Mental Health diagnosis.*

c *Reflects the number and percentage of participants answering “more [than before lockdown]” or “most of the time” [during lockdown] or “strongly agree.”*

* *p < 0.05 (for χ^2^ tests)*.

**Table 3:** Parenting during COVID-19 lockdown.

	**Non-student Parents (*****N*** **=** **13)**		**Student Parents (*****N*** **=** **14)**		**PMHD**^****a****^ **students (*****n*** **=** **8)**		**NPMHD**^****b****^ **students (*****n*** **=** **6)**	
	***n***	***%***	***n***	***%***	***n***	***%***	***n***	***%***
**Parenting**^**c**^								
*Difficulties*								
Felt overwhelmed by childcare responsibilities [some or most time]	8	61.5	11	78.6	6	75	5	83.3
Been anxious about their child's education	6	46.2	6	42.9	2	25	4	66.7
Struggled with routine and structure [some or most time]	9	69.2	11	78.6	7	87.5	4	66.7
Hard to juggle childcare and work [some or most time]	10	76.9^*^	11	78.6	6	75	5	78.6
Struggled to cope with managing their children [some or most time]	6	46.2	9	64.3	6	75	3	50
Not had enough time to themselves [most of the time]	11	84.6^*^	5	35.7	4	50	1	16.7
*Strengths*								
Children had enjoyed being at home [some or most of the time]	11	84.6	14	100	8	100	6	100

a *PMHD, Pre-existing Mental Health diagnosis.*

b *NPMHD, Non Pre-existing Mental Health diagnosis.*

c *Reflects the number and percentage of participants answering some or most of the time combined.*

* *p < 0.05 (for χ^2^-tests)*.

As a proxy measure of mental health vulnerability participants were asked the following question “Have you ever been diagnosed with a mental health condition?” thus enabling to distinguish between two categories of participants: those with pre-existing mental health diagnosis and those without.

In addition, *qualitative data* from participants were obtained *via* two open ended questions in the survey, which asked participants to look back over the lockdown period, and report in their own words what have been their personal strengths and difficulties/challenges.

Primary research participant group were adult individuals aged 18 years and over living in the UK during the COVID-19 pandemic and were recruited with support from Relate, through opportunity sampling, including social media advertising as well as snowball techniques. Research data were collected *via* an anonymous on-line survey and managed using the Online surveys, a secure, web-based software platform hosted by Jisc.ac.uk.

Ethical approval was granted for all aspects of the study by the College of Business, Psychology & Sport Research Ethics Panel at the University of Worcester. Potential participants were directed to study information *via* a weblink; those who wished to take part (after being provided with full information about the study were required to confirm that they met eligibility criteria (being aged 18 and over) and to voluntarily consent to take part. Participants then proceeded to complete an online survey.

### Analysis Strategy

In this report the aim was to compare female students who reported a *pre-existing mental health diagnosis (PMHD sub-group)* prior to the pandemic across a number of self-ratings with those who *did not have a pre-existing mental health diagnosis (NPMHD group)*. For comparison purposes, a random case-matched sample of non-students was extracted from the *Families Un-Locked* study data, matched on gender, pre-existing mental health diagnosis, couple relationship, and parent status.

Given the small sample size which limits the extent of statistical analysis, the reporting of results is cautious, identifying emerging associations and aiming to inform future research, and tentatively propose implications for student support. Chi-square test (χ^2^) were performed to test for significant (a *p* < 0.05 was considered statistically significant) associations between coping reported by participants with a pre-existing diagnosis, compared to participants who did not and between student and non-student participants. Qualitative data derived from the free text in the survey responses were used to corroborate the emerging associations in findings and further explored for emergent coping themes, across responses from participants with PMHD to NPMHD.

## Results

### Demographic Variables

The 36 women students in the analysis sample were aged between 18 and 64 (m = 39.89, SD = 13.97, mode 18, 34). The age distribution was broad which can be explained by the fact that data were extracted from a larger study which did not target students in particular.

Nevertheless, this offered the opportunity to explore a non-traditional group of women students, the majority being in a committed relationship and just over a third (37%) were parents of children aged under 18 and living in their household (between 1 and 4 children). Over a third (39%) of the women reported a pre-existing mental health diagnosis and its prevalence in this group is higher than that reported in the 2020 University Student Mental Health Survey (26.6% reported in Pereira et al., 2020).

### Comparing Student vs. Non-students

Between group analyses on coping revealed no significant differences between students and non-students. When couples (*n* = 65) were compared across the two samples on the impact of the lockdown on their couple relationship, more non-students than students reported that they felt tension/strain in their relationships (χ^2^ = 10.842, df = 3, *p* = 0.013). In addition, more non-students than students strongly disagreed with the statement “Lockdown has been a positive experience for us” (χ^2^ = 16.755, df = 3, *p* = 0.001) and strongly agreed with the statement “We worried a lot about the pandemic, which is causing tension in our relationship” (χ^2^ = 8.955, df = 3, *p* = 0.03).

A similar trend was apparent when students and non-students were compared on their parenting and managing their children during lockdown: more non-student (than student) parents reported difficulty to juggle childcare and work (χ^2^ = 7.640, df = 2, *p* = 0.022), supervising their children or “keep a constant eye on what my children are doing” (χ^2^ = 6.639, df = 2, *p* = 0.036) and having to keep children busy or “entertaining or playing with them” (χ^2^ = 6.794, df = 2, *p* = 0.033) and overall, more reporting not having enough time for themselves (χ^2^ = 7.056, df = 2, *p* = 0.029). Collectively, these findings tentatively suggest that more non-student couples and parents felt under pressure during the lockdown due to balancing their work roles and those of partner/parent.

Henceforth the results are reported as comparisons between students and non-students where relevant, as well as within sample comparisons and between the sub-group of students with pre-existing mental health diagnosis (PMHD subgroup *n* = 14 and those without (NPMHD subgroup *n* = 22).

### The Impact of the Lockdown on Women Students' Pre-existing Mental Health Difficulties

The majority (71%) of women students who reported having a pre-existing mental health diagnosis, which pre-dated the first lockdown, reported that their mental health had been *made worse* by the lockdown. This finding on the negative impact of the lockdown on the mental health is concurrent with existing research with both students (Hewitt, 2020) and the general population (Jia et al., 2020; Kousoulis et al., 2020; Polizzi et al., 2020; Fancourt et al., 2021) samples of individuals with pre-existing mental health diagnoses.

### Coping With the Lockdown

As illustrated in Table 1, data suggest a picture of women students who have had to adapt with flexibility to the changes over the COVID-19 lockdown period.

#### Loneliness

Around a third of students (34%) reported that they felt lonely most of the time during lockdown and missed seeing family (30%) and friends (42%) most of the time. Significantly more students with PMHD reported feeling lonely most of the time during lockdown than non-students with PMHD (χ^2^ = 6.552, df = 2, *p* = 0.038). However, there were no significant differences found between students with or without pre-existing mental health diagnoses.

These results echo findings by Bu et al. (2020) that being a student was a risk factor for higher loneliness than non-students; and that women and people with pre-existing mental health diagnoses and students were significantly more likely to be in the highest loneliness category. Women reported more socially supportive relationships prior to lockdown (Bu et al., 2020) making it possible that the sense of isolation was more acute when this support was less accessible. Loneliness was also reported to be a risk factor which negatively impacted coping (Varma et al., 2021).

#### Anxiety

Just over a quarter of students (28%) reported feeling anxious *most of the time* during the lockdown; and just under a third had trouble relaxing *most of the time*; however, no significant differences were found in reported feelings of anxiety between those with pre-existing mental health diagnoses and those without.

This finding is in tandem with reports that there has been a general decline in well-being throughout the population due to the circumstances (Jia et al., 2020; Polizzi et al., 2020) and data collated by surveys on students (Save the Studen, 2017; National Union of Students, 2020; Office for National Statistics, 2020). Also, pre-pandemic adults with pre-existing mental health diagnoses reported worse levels of mental health but this gap did not widen during the pandemic (Fancourt et al., 2021).

### Coping Behaviours

A range of coping behaviours during the lockdown were reported as illustrated in Table 1. Among positive coping strategies, around 40% of students reported going out *more* (for exercise or walks, as permitted at the time) and spending *more* time connecting with family and friends remotely. Around a third spent *more* time on hobbies and just over a quarter (28%) exercised *more*. A third reported drinking more alcohol than before the lockdown and 40% eating more which are negative coping strategies raising the risk for mental and physical health. This mixture of positive and negative coping is consistent with trends described in research elsewhere (Fluharty and Fancourt, 2020).

Overall, more students with PMHD reported avoidant coping and negative emotion focussed coping during lockdown: eating more during lockdown (χ^2^ = 7.387, df = 2, *p* = 0.025), having had trouble relaxing during lockdown (χ^2^ = 5.653, df = 2, *p* = 0.05), and not feeling at all positive about the future (χ^2^ = 6.085, df = 2, *p* = 0.048). The association persisted when PMHD students were compared with PMHD non-students: significantly more students with PMHD reported not feeling positive about the future than non-students with PMHD (χ^2^ = 6.328, df = 2, *p* = 0.042), indicative of a level of hopelessness in the students with mental health issues. This concurs with findings from a study of Polish students that students with PMHD can be particularly vulnerable and require urgent support (Rogowska et al., 2020).

Adults with pre-existing mental health diagnoses have been found to be more likely to use avoidant coping during the pandemic (Fluharty and Fancourt, 2020) and these coping behaviours have been linked to increased psychological distress (Dawson and Golijani-Moghaddam, 2020) in a cycle of ineffective coping which can be harmful to physical health (increased substance use) and psychological health (Gurvich et al., 2020).

Students with PMHD also reported (*via* the free text items) specific behaviours indicative of avoidant coping, for example: “*Staying busy”;“plodding on and allowing the doldrums to pass,”* both suggesting emotional disengagement; and overall less emotional coping such as:

“*Feeling very tired, having very little motivation, feeling anxious and depressed”*

“*Loneliness and realising how isolated I am compared to my family and friends”*

Although not reaching statistical significance, students with NPMHD reported engaging more in positive coping during lockdown, such as spending more time on hobbies and exercising. These two types of activities were allowed under lockdown conditions, suggesting that these students engaged in positive adaptive behaviour by making the most of what was available to them, and generally re-framing the restrictions in positive terms such as the quotes below illustrate:

“*I've been able to build on strong friendships, resilience, common sense in the face of changing advice and guidance.”*

“*Routines, generally still eating healthily and exercising, maintaining a strong relationship with partner.”*

“*Spending time with my parents who I don't usually see as often when I'm away for uni helped.”*

#### Social-Focused Coping

Because social interactions were severely curtailed during the lockdown, individuals who habitually relied on social-focused instrumental coping were obviously unable to draw on such strategies, as illustrated by students' testimonies about their personal challenges during the lockdown:

“*Not being able to go to school/college, having to work remotely. Feeling isolated. Worried about the effects of isolation on my children- not seeing their friends, not knowing how long this was going to last, Not knowing which information to believe as it kept changing.”*

The quote above also illustrates the confusion created by the conflicting information provided at times regarding the virus and the pandemic; and a student reported disengaging from media as a “strength” and positive coping strategy:

“*Staying calm, stopping social media and media connectivity, accepting*.”

Problem focused coping focusses on the source of stress and attempts to deal with it (Stanisławski, 2019). Control over the situation was severely curtailed as were opportunities to affect it. This choice demonstrates one area of managing media contact and exerting appropriate control where it was possible. They also state “accepting” which is a positive emotional focussed strategy based on acceptance of the reality of the situation (Fluharty and Fancourt, 2020).

#### Couple Relationships and Parenting

Faced with the unavailability of the social support from direct contact with friends, some individuals turned for support to family relations within household, with both partner relationships and parenting featuring among reported strengths and challenges. Participants perceived impact of the lockdown on their couple relationship and parenting are summarised in Tables 2, 3, respectively.

Significant differences in coping between students in couple relationships (*N* = 29) with PMHD (*n* = 11) and without (NPMHD, *n* = 18) were detected in our data (Table 2), with those with PMHD reporting eating more (χ^2^ = 4.974, df = 1, *p* = 0.026) and less feeling positive about the future (χ^2^ = 6.626, df = 2, *p* = 0.036) during the lockdown, a trend similar to that in the whole sample of students. However, there were no significant differences in reported impact of lockdown on their couple relationships or parenting. Within the student parents subgroup (*n* = 14), there were no significant differences between those with PMHD and without (NPMHD) in terms of their coping or managing children (Table 3).

The majority of participants were in a committed relationship and these featured among the strengths mentioned:

“*Maintaining a strong relationship with my partner”*“*Strong relationships with partner and friends”*

Parents with young children were identified as a vulnerable group in previous studies, suggesting that their mental health was negatively impacted with anxiety being higher but depression lower than in non-parents (Niedzwiedz et al., 2020; Pierce et al., 2020). This suggests that the ways in which parenting intersects with wellbeing are complex and nuanced: for some parenting acts as a protective factor, offering distraction and a sense of clear priorities, while for others, a pressure and additional concerns about children's well-being.

However, in our sample there were no significant differences between student parents and those who were not parents in terms of their reported coping and mental health impact. Moreover, student parents in our study (*n* = 13) described their role as parents and interactions with their children as both a strength and difficulty, as the quotes below from three different parents illustrate:

Parent 1:

[Strength] “*Have home schooled successfully and my daughter has made great progress…. Reassuring my daughter when she has been anxious and sad about lockdown”*

[Difficulty] “*Feeling overwhelmed with solo parenting and feeling like I was doing a bad job of it.”*

Parent 2:

[Strength] “*Providing children with love”*

[Difficulty] “*Guilt that my children were not having enough things to do. Lack of energy to provide entertainment for children”*

Parent 3:

[Strength] “*I have kept boundaries with my children, forcing exercise and fresh air. Specific screen times, despite daily conflict when I do so. That's been my major stress!”*

[Difficulty] “*Homeschooling, relationship with my teenage son. Setting a daily routine for my children. Battles with xbox/screen time for my children.”*

### Reflections on Individual Strengths and Challenges in Navigating the Pandemic

Two items on the survey collected qualitative data detailing what participant's felt were their strengths and personal difficulties or challenges over the lockdown period. When identifying strengths, participants covered a variety of factors, including but not limited to practical skills (such as online shopping), spending more time on their hobbies, and personal traits (such as patience). Parents reported being focused on keeping children healthy and supporting their well-being. Describing challenges or personal difficulties participants reported struggling with a range of issues: family and relationship breakdown stressful social interactions on new norms (mask wearing and social distancing), negative coping behaviours (overeating, lack of self-belief) areas of life which have been impacted such as future employment concerns.

A trend became apparent in the style of language used. When reporting their strengths, students with pre-existing mental health diagnoses tended to describe things they had done to cope rather than internalised, personal strengths. For example, one student described as her strength:

“*Able to focus more on study without worrying about travelling and attending courses in the room.”*

And the extracts below described actions:

“*Carrying on with my work. Trying to bounce back through depression and other mental health issues”*

“*Have carried on going, nearly finished my PhD”*

By contrast, students who did not have a pre-existing mental health diagnosis, described their strengths during the lockdown as personal abilities, qualities/traits or personal resources such as money and pets:

“*My ability to be comfortable in my own company. My ability live small life. My determination to see things through. My ability to comply with rules. My ability to accept things as they are.”*

“*I am optimistic I like to have fun and connect with others I have lots of friends…. I volunteer I support others…. We have enough money I have pets….”*

Although they also identified behaviours as strengths, these appeared to be more internalised by clearly attributing the behaviour to themselves:

“*Looking after my children. Keeping up my studies.”*

“*Learning that I am resilient. Listening to my children and sharing their worries and concerns.”*

The internalised language and self-attribution of strengths in students without pre-existing mental health diagnoses suggests a sense of self-efficacy and perceived control which are processes underpinning resilience (Dvorsky et al., 2020). Students with pre-existing mental health diagnoses reported challenges such as social isolation and lockdown preventing access to support, which have been identified as likely to add additional pressure on those with vulnerabilities (Jia et al., 2020; Kousoulis et al., 2020):

“*Not being able to access my normal coping mechanisms”*

“*being isolated from family and friends”*

“*Being away from my mum for four months as we are really close and usually see each other weekly”*

They also identified difficulties suggesting negative coping behaviours or indicative of struggles to maintain mental well-being.

“*Motivation to cook, shower, get out of bed. Wanting to do anything that I used to enjoy eg reading painting, doing my makeup.”*

“*Feeling very tired Having little motivation. Feeling anxious and depressed. Having very little time on my own”*

“*Over eating Under eating Lack of exercise Worse mental health”*

“*Depression Shielding Lack of motivation Boredom Anxiety and family difficulties”*

However, they also gave examples of positive coping behaviours, such as engaging in activities and re-framing.

“*I kept myself busy by learning how to bake which lifted the mood in the house”*

“*Finding positives in the situation”*

Engaging in substitute activities can be conceptualised as an avoidant coping strategy, however, in the lockdown context of home confinement, “keeping busy” was an adaptive strategy, illustrating the context-dependent value of coping. Coping strategies that can be adaptive in certain contexts and maladaptive in others (Stanisławski, 2019).

When identifying strengths, students without mental health diagnoses reported positive coping behaviours, such as:

“*Making times for my hobbies to look after my mental health”*

“*Being able to relatively quickly adjust to the new situation. Still kept in contact with family and friends.”*

“*Family Self-reflection Mindfulness/Yoga Exercising more”*

Students without mental health diagnosis outlined more positive coping which in turn facilitated better mental health outcomes, leading to better adaptation during the lockdown. Whereas, students with mental health diagnoses reported more avoidant coping which is usually associated with poorer mental health (Gurvich et al., 2020). This is unsurprising perhaps as the choice of coping is influenced by personal circumstances and histories.

Social isolation was identified as a difficult by both students with or without mental health diagnoses as these quotes illustrate.

“*Not being able to see family. Not being able to visit my country of origin.”*

“*Not being able to go to school/college. Having to work remotely. Feeling isolated”*

“*Not being able to see my boyfriend for months”*

Perhaps it is unsurprising that both groups identified isolation as a main difficulty/challenge during the lockdown, as social distancing and lack of household mixing were core strategies for controlling the spread of the virus.

Only students without pre-existing mental health diagnoses mentioned pressures of academic work and logistics of student life—for example:

“*Working at home full time. Only having online contact with student peers and my supervisors. I've missed the culture and community of my campus.”*

“*Having to leave my university course prematurely and missing my friends there.”*

“*Motivation to complete dissertation”*

“*Anxiety Degree worries Low mood”*

“*Trying to complete my uni assignments when classes were cancelled.”*

Among students with pre-existing mental health diagnoses, academic struggles were not specifically mentioned, and this could be due to other difficulties taking precedence for them during the lockdown, such as managing their mental health. However, the small sample size limits the conclusion that can be drawn from current data, and this issue warrants further investigation.

## Discussion

Isolation has been a commonly reported feature of lockdown (Jia et al., 2020; Kousoulis et al., 2020; Rogowska et al., 2020; Fancourt et al., 2021). With a student community now geographically separated across the UK and abroad, creating a sense of community is an incredibly difficult challenge. Support for and identification of students who feel isolated through an automated check in, could then enable institutions to follow up responses that request this. Institutional logistics determine the practicalities, but the importance of connection has been demonstrated throughout the pandemic. Students' mental health has been deleteriously affected (Hewitt, 2020) and many will need support to succeed in continuing their studies. Vulnerable students are already less likely to succeed (Lawrence et al., 2006), thus supporting their continuation of studies makes this endeavor beneficial for both the student and the institution. Provision and support are likely to be already limited (Macaskill, 2013) and the sector needs to continue to expand both. Facilitating contact and creating opportunities for students to connect with their peers for both academic purposes and socially, go some way to address the issue of isolation which was reported by students in this sample. The University student community has been disrupted significantly, and understanding how this can be addressed by institutions is a starting point for policy and planning. This study identifies some vulnerabilities and challenges reported by women students. Next steps in policy planning will be determined by budget constrictions, but where there is opportunity this should be made a priority (Universities United Kingdom, 2018).

Our findings suggest that everyone including female higher education students had to make many adjustments during the first lockdown in 2020. It is reasonable to argue that this situation has continued for the remainder of the year to December 31, 2020. Higher education institutions have had to shift to remote delivery and most have done so with varied success (Crawford et al., 2020). Transitions are complicated to navigate at individual and institutional levels (Denovan and Macaskill, 2016). A return to campus teaching may be seen as desirable, but this assumes shared attitudes with regards to risk and the positive nature of in person teaching and study. Flexibility when planning for campus returns, would provide students who have found remote learning positive, with an option to continue without institutional pressure. It should also be considered that familial and financial circumstances may have drastically changed during the pandemic. Bereavements, health issues and unemployment have occurred unexpectedly to many people and disproportionally impacted women (LeanIn, 2020; Power, 2020).

Students are not a homogenous group but there is evidence to suggest that they frequently report similar concerns. The data reported here are from women students the majority of whom are in committed relationships and over a third are also parents. Women students who have children living at home reported finding their role as parent both fulfilling, supportive and challenging. Childcare has consumed more time for many parents in the UK this year following the closure of schools. Characterisation of parenting as a situationally significant factor is justified (Daly et al., 2020) and parental responsibility impacts daily functioning. Flexibility in access to content enables parents to fit their studies around childcare. A (partial) closure of schools in the UK beginning in January 2021 is further evidence that the pandemic's impacts on parents and families is likely continue.

Coping styles for students with pre-existing mental health diagnoses, showed an emerging trend toward more negative coping behaviours and fewer positive coping behaviours. This is supported by trends identified elsewhere; being unable to access usual support systems, affected those with pre-existing mental health diagnoses in the general population throughout 2020 (Jia et al., 2020; Kousoulis et al., 2020; Fancourt et al., 2021). Students with no mental health diagnoses reported more positive coping behaviour and specified details related to positive coping in response to the open question about strengths. Identifying students who are particularly vulnerable will be key to supporting their continuing education. Engagement with university mental health services was reported to be low (20%) by students (National Union of Students, 2020) and such barriers need to be examined and addressed by universities in the future.

### Limitations and Strengths

Findings from this study should be interpreted in light of the limitations derived from the small sample size, and the use of newly designed (non-standardised) items to capture participants' (retrospective) reporting of coping and feelings during the lockdown. The key strength of this study is the focus on women students with and without pre-existing mental health diagnoses, which is an under researched group. Nevertheless the sample of women students had a higher prevalence of self-reported diagnosed mental illness (39%) relative to the general population of students (26.6%, Pereira et al., 2020), thus limiting the generalisability of these results. Importantly, this study provided the opportunity to hear first-hand accounts from students about their coping during the lockdown and reflections on personal strengths and challenges. These contribute to a deeper understanding of the impacts of the COVID-19 lockdown.

## Conclusion

Lockdown required adaptation and appropriate coping strategies to be employed to deal with novel stressors and the “new normal.” The risk factors linked to increased psychological distress and less adaptive coping are: being female, a young adult, student, having a pre-existing mental health diagnosis and being a parent, amongst others (Fluharty and Fancourt, 2020; Gurvich et al., 2020; Kousoulis et al., 2020; Niedzwiedz et al., 2020; Fancourt et al., 2021). This report examined a group of female students of various ages, with 39% reporting a pre-existing mental health diagnosis, which is a high prevalence of self-reported diagnosed mental illness relative to the general population of students. Students with a pre-existing mental health diagnosis reported higher levels of loneliness compared to non-students with a pre-existing mental health diagnosis. Students with pre-existing mental health diagnosis reported more avoidant coping and negative emotional coping than students without a diagnosis. Finally, parenting intersects with well-being and stress as both a protective and risk factor.

Further research into vulnerable student groups is needed to understand how this complex picture can be used by HE institutions to provide targeted support and to inform both preventative and targeted interventions. Last but not least: while acknowledging that the disruption for all students throughout 2020 has been significant, it is also important to note that while some students found themselves struggling, others found ways to cope well. Recognising and supporting the steeling effect of coping with adversity is the foundation of building resilience, which may in turn inform preventative interventions.

## Data Availability Statement

The raw data supporting the conclusions of this article will be made available by the authors, without undue reservation.

## Ethics Statement

The studies involving human participants were reviewed and approved by The College of Business, Psychology and Sport Research Ethics Panel (BPSREP), University of Worcester. The patients/participants provided their written informed consent to participate in this study.

## Author Contributions

GM designed the study from where the data set was extracted, undertook analyses, and drafted the manuscript. GT has undertaken the literature review, analysed qualitative data, and drafted the manuscript. Both authors have reviewed and approved the final manuscript.

## Conflict of Interest

The authors declare that the research was conducted in the absence of any commercial or financial relationships that could be construed as a potential conflict of interest.
